# Dr. Girdlestone, on the Use of Mercury in Dropsy

**Published:** 1802-05

**Authors:** T. Girdlestone

**Affiliations:** Yarmouth


### To the Editors of the Medical and Phyſical Journal.

GENTLEMEN,

IF you think this Paper worth inſerting in your uſeful Journal, it is much at your ſervice. From your's, &c.

*Yarmouth, Feb.* 3, 1802. T. GIRDLESTONE, M. D.

---

THOSE who are acquainted with the opinions and practice which prevailed in the time of Sylvius Deleboe, and Van Helmont, know that theſe authors, and many of their contemporaries, depended on a mercurial ſalivation for the cure of dropſies. From Gremb's *Arbor Integra, et Ruinosa Hominis*, a work printed in 1657, and reckoned by Ettmuller to be the book which beſt explains the doctrines of Van Helmont, the following extracts are taken.

"Intentio curandi hydropem ex Helmontio habetur. 1<sup>ma</sup>. ut devia qualitas, quæ renem a ligatura et abdomen ab aqua liberat, dematur, tum ren errorem cognoſcet et abſorbet ad ſe laticem: itaque hydropem eſt ſerum renum pertinacem ſolvere, prout ſolvere cruorem coagulatum eſt ſolvere cauſam occaſionalem; hinc hydropem non juvant hydrogoga ſed mercurius."

. . . . . . . . . . . . .

"Uſque dum ſalivatio oris ſuperveniat." p. 118, 119, De defectibus Hepatis.

"Aſthma inducit hydropis quædam ſpecies, ubi hepar ſupra modum excreſcit, diaphragma et pulmones comprimet, aliquando reliquæ partes tabe conſumantur, aliquando tamen præter excreſcentiam hepatis etiam venæ pulmonum turgent. Helmontius ſcribit creſitivam illam vim per mercurium enecandam eſſe." P. 157, De Defectibus Pulmonum.

I have been led to quote the above paſſages becauſe the late ingenious Dr. Withering has ſuppoſed, that the hydrothorax, when unaccompanied with aſcites may be generally cured by the diuretic effects of digitalis. But I have ſeen many exceptions to this remark, and witneſſed many caſes of hydrothorax, where the morbid effects of digitalis had nearly taken away the life of the patient without relieving the difficulty of breathing; and where this ſymptom was as much occaſioned by the action of the diaphragm being impeded by a diſeaſed growth of the liver, as by the effuſion about the lungs or heart. To attempt the cure of ſuch a caſe by digitalis, is only to exhauſt the ſtrength of the patient ſo far by attacking the effects rather than the cauſe of his diſeaſe, as to leave him often afterwards too feeble to hope for any remedy from other medicines. In fuch cafes, I prefume, the practice recommended by Van Helmont, &c. ought always to be tried before any portion of digitalis be given. And in every cafe of hydrothorax there will probably be lefs wafte of ftrength if a flight ptyalifm be induced by mercury before any of the digitalis be adminiftered; becaufe then dofes of five or ten drops of the tincture of this medicine will often effect, in two or three days, what double the quantity taken for fix or feven days could not do, if no mercury had firft pervaded the habit.

More than a year ago, a gentleman upwards of ninety-two years of age, and whofe habits throughout life had been directed by the moft exact regard to temperance in eating, drinking, and exercife, became the patient of a refpectable practitioner in this town for an anafarca and hydrothorax: The digitalis was given to him in tincture, and perfifted in for feveral days, after a naufea, lofs of fpirits and ftrength had been induced, without producing any diuretic effects or diminution of the dropfical fymptoms.

It was not without difficulty that he was afterwards prevailed on to return to the trial of any fort of medicine. He at laft fubmitted to the fwallowing of fmall dofes of calomel and opium every fix hours. Thefe medicines, in a week or ten days, diminifhed the anafarcous fymptoms, and by a perfeverance in them, with the addition of bark and fteel, he re-eftablifhed his health, and is now perfectly well.

The patient of another refpectable practitioner, a lady confiderably above eighty years of age, and whofe habits had been equally temperate as thofe of the preceding patient, laboured under anafarca and hydrothorax. The digitalis was given to her, and it conftantly produced diuretic effects; but it increafed the anafarcous fymptoms till a purple blufh came all over the upper and lower extremities, and the perfeverance in this medicine increafed the irritability of the mind to fuch a degree, that fhe became delirious. By leaving off the digitalis the delirium in three or four days fubfided. But neither bark with mineral acids, cream of tartar, nor any other medicine, being able to prevent the efcape of the red parts of the blood through the exhalents, fhe did not live above ten days after the difcontinuance of the digitalis. I do not know whether it has occurred to other practitioners, but many cafes have come under my obfervation, where the digitalis feemed to have increafed very much the irritability of the mind. An elderly lady with hydrothorax and anafarca, who was cured by only forty-five drops of the tincture of digitalis in dofes of five drops, for four days after the laft dofe remained perfectly infane. She continued feveral feveral months without any return of the hydrothorax; but the horror with which the effects of the digitalis had infpired her againft that medicine, made her prefer the re-appearance of her difeafe to her remedy, or the trial of any other medicine, till fhe died.

It is of great confequence in giving fo powerful a medicine as digitalis to aged people, that not a fingle dofe be repeated after the breathing has been fufficiently relieved. I have feen this medicine continued till a fort of paralytic tremor had been induced over almoft all the mufcles of the body, either through inattention or the belief that the diuretic effects had leffened; and where the leffening of the quantity of the urine was only becaufe the anafarcous effufion was nearly all abforbed and dif-charged; and where bark, fteel, or zinc, with fmall dofes of opium, might fooner have been given with the beft effects. By attention to thefe points I think I have been able to prolong the lives of fome patients with hydrothorax to an unufual length. The following is the cafe of my firft patient in this part of the country with this difeafe. Ten years ago a very diftinguifhed fcholar, became my patient for an afcites, hydrothorax, hydrops pericardii, and anafarca. He was given mercury till a flight falivation was induced, then fquills till the afcites, hydrothorax, and anafarca difappeared; and afterwards with bark and fteel his ftrength was recovered. He has fince fuffered two or three returns of the hydrothorax and anafarca, which have been each time taken away by inducing a flight ptyalifm by mercury, and afterwards giving a few drops of the tincture of digitalis for a day or two before bark and tonics were refumed. He is now cheerful in the eighty-firft year of his age, breathing feemingly better than he did ten years ago: But as the flender inter-rupted pulfe which he had when I firft attended him, never was entirely taken away, I prefume that an hydrops pericardii to a certain degree has always remained.

During the whole ten years he has never been permitted to go above a week or ten days without taking, over night, a grain or two of calomel.

The more urgent fymptoms of fyphilis being blended with other complaints, have probably led to the ufe of mercury in many difeafes. A few of the following remarks which I have met with in turning over the pages of a variety of medical authors, may be new to fome readers.

Poterius obferves, that thofe who have rubbed in mercurial ointment, or thofe who dig in the mercurial mines, are fcarcely ever afflicted with fevers. He, Piens, Ballonius, Willis, Hartman, Ettmuller, and many others, cured all obftinate inter-mittents by a mercurial falivation, as Sir George Baker did an intermittent intermittent patient who laboured under ſiphilis. The catholicon of Van Helmont in fevers was a ſecret preparation of mercury; and Dolæus, the friend of our great Sydenham, in his Encyclopœdia on Fevers, has the following recipe and remark.

"℞. Mercurii dulc. gr. xv. Antimon. diaphoret. gr. vj. M.

"Magni venditatur in febribus continuis et intermittentibus panacea illa duplicata quæ me raro fefellit ad ℈j. cum oculis cancrorum exhibita in liquore conveniente." Chap. i. p. 27, de Febribus in genere Encyclopœdiæ Medicæ, Johannis Dolœi, Francofurte, 1691.

From the manner in which Ariſtotle, Galen, Oribaſius, Paulus Ægineta, Ætius, Actuarius, Nicander, Dioſcorides, Pliny, &c. have taken notice of mercury, it is ſuppoſed to have been ſeldom uſed internally before the fifteenth century. But as Auſonius lived in the fourth century, and is ſaid to be the ſon of a phyſician, we can hardly read the following Epigram of his, which Simon Paulli has quoted in his Digreſſio de Febribus Malignis, without believing that either through his father, or ſome other means, this poet had been acquainted with the internal uſe of mercury as a medicine.

### Epigram X.

Toxica Zelotypo dedit uxor mæcha marito:

  Nec ſatis ad mortem credidit eſſe datum.

Miſcuit argenti lethalia pondera vivi

  Cogeret ut celerem vis geminata necem.

Dividat hæc ſi quis, faciunt diſcreta venenum,

  Antidotum ſumet, qui ſociata bibet.

Ergo inter ſeſe dum noxia pocula certant

  Ceſſit lethalis noxa ſalutiferæ:

Protinus et vacuos alvi petiêre receſſus

  Lubrica dejectis qua via nota cibis.

Quam pia cura Deum! prodeſt crudelior uxor,

  Et quum fata volunt, bina venena juvant.

